# Predictors of caregiver burden in aged caregivers of demented older patients

**DOI:** 10.1186/s12877-021-02007-1

**Published:** 2021-01-14

**Authors:** Chia-Fen Tsai, Wei-Shen Hwang, Jun-Jun Lee, Wen-Fu Wang, Ling-Chun Huang, Li-Kai Huang, Wei-Ju Lee, Pi-Shan Sung, Yi-Chien Liu, Chih-Cheng Hsu, Jong-Ling Fuh

**Affiliations:** 1 Department of Psychiatry, Division of Geriatric Psychiatry, Taipei Veterans General Hospital, Taipei, Taiwan; 2 Department of Psychiatry, Tungs’ Taichung Metroharbor Hospital, Taichung, Taiwan; 3 Department of Neurology, Kaohsiung Chang Gung Memorial Hospital and Chang Gung University, College of Medicine, Kaohsiung, Taiwan; 4 Department of Information Management, National Sun Yat-sen University, Kaohsiung, Taiwan; 5 Department of Neurology, Changhua Christian Hospital, Changhua, Taiwan; 6 Department of Holistic Wellness, Ming Dao University, Changhua, Taiwan; 7 Department of Neurology, Kaohsiung Municipal Ta-Tung Hospital, Kaohsiung Medical University, Kaohsiung City, Taiwan; 8 Department of Neurology, Neurological Institute, Taipei Veterans General Hospital, Taipei, 112, Taiwan; 9 Department of Neurology, Shuang Ho Hospital, Taipei, Taipei Medical University, New Taipei City, Taiwan; 10 Graduate Institute of Humanities in Medicine, Taipei Medical University, Taipei, Taiwan; 11 The PhD program for Neural Regenerative Medicine, College of Medical Science and Technology, Taipei Medical University, Taipei, Taiwan; 12 Neurological Institute, Taichung Veterans General Hospital, Taichung, Taiwan; 13 Faculty of Medicine, National Yang-Ming University Schools of Medicine, Taipei, Taiwan; 14 Department of Neurology, National Cheng Kung University Hospital, College of Medicine, National Cheng Kung University, Tainan, Taiwan; 15 Neurological Center of Cardinal Tien Hospital, Taipei, Taiwan; 16 Fu Jen University School of Medicine, Taipei, Taiwan; 17 Institute of Population Health Sciences, National Health Research Institutes, Zhunan, Taiwan; 18 Division of General Neurology, Neurological Institute, Taipei Veterans General Hospital, Taipei, Taiwan

**Keywords:** Caregiver burden, Oldest-old, Zarit burden interview, Older caregiver

## Abstract

**Background:**

Dementia in the oldest-old is projected to increase exponentially as is the burden of their caregivers who may experience unique challenges and suffering. Thus, we aim to investigate which factors are associated with older caregivers’ burden in caring demented outpatients in a multicenter cohort.

**Methods:**

Patients and their caregivers, both aged ≧65 years, in the National Dementia Registry Study in Taiwan (T-NDRS) were included in this study. Caregiver burden was measured with the short version of the Zarit Burden Interview (ZBI). The correlations between the ZBI scores and characteristics of caregivers and patients, including severity of dementia, physical comorbidities, instrumental activities of daily living (IADL), neuropsychiatric symptoms assessed by the Neuropsychiatric Inventory (NPI), and family monthly income, were analyzed.

**Results:**

We recruited 328 aged informal caregiver-patient dyads. The mean age of caregivers was 73.7 ± 7.0 years, with female predominance (66.8%), and the mean age of patients was 78.8 ± 6.9 years, with male predominance (61.0%). Multivariable linear regression showed that IADLs (β = 0.83, *p* < 0.001) and NPI subscores of apathy (β = 3.83, *p* < 0.001)and irritability (β = 4.25, *p* < 0.001) were positively associated with ZBI scores. The highest family monthly income (β = − 10.92, *p* = 0.001) and caregiver age (β = − 0.41, *p* = 0.001) were negatively correlated with ZBI scores.

**Conclusions:**

Older caregivers of older demented patients experience a higher care burden when patients had greater impaired functional autonomy and the presence of NPI symptoms of apathy and irritability. Our findings provide the direction to identify risky older caregivers, and we should pay more attention to and provide support for these exhausted caregivers.

## Background

Dementia is characterized by irreversible and progressive impairments in cognition, behavioral function and activities of daily living (ADL). The growing numbers of people affected by dementia, with the number expected to double every 20 years worldwide, makes caring in dementia a global public health issue [1]. Because the symptoms of dementia interfere with patient function and increase their dependency, patients with dementia require more support in daily living and long-term care from their caregivers, which often results in substantial financial and health distress of their caregivers [2]. The obligation to care for dementia patients is often shifted to their original family, mainly the spouse, children or siblings of the patients. Many of them have to cut back on work to take the role as caregivers and face additional expensive medical services. Dementia patient caregivers encounter extra household expenditure, which has been shown to be associated with their caring role [3]. As family is expected to be the primary source of care going forward, especially in Asian populations, understanding the burden suffered by family caregivers of dementia patients is of great importance.

Dementia caregiving has negative associations with the caregiver’s physical and mental illness [4–6]. Previous research has found that various factors, such as neuropsychiatric symptoms (NPS) and abnormal behaviors of demented patients, more severe dementia severity, higher functional dependence, cognitive impairment, low level of education and family income, and impaired health status of the caregivers are significantly associated with higher caregiver burden [7, 8]. Although studies have tried to investigate the factors associated with caregiver burden, there is a lack of research focusing on older caregivers of patients with dementia. The age of most caregivers in past studies is often mixed, ranging from 50 to 65 years old [9–12].

Taiwan has entered the stage of an aged society as people over 65 years old accounted for more than 15.3% of the country’s total population [13], and this group includes a large number of demented patients and their caregivers. However, the increase in dementia in the older adults coincides with a dramatic decline in the potential support ratio, namely, the number of persons aged 20–64 per person aged 65 or older [12]. According to the population projections reported by the National Development Council of Taiwan, the potential support ratio is predicted to drop from 5.9 in 2015 to approximately 2.7 by 2030 [14], with similar declines expected in most countries worldwide [12, 15]. In recent years, there has been an increase in the number of older people who have become caregivers for their elderly relatives. There is a need to examine the social context in which an older individual must take care of a centenarian one with dementia, although this emerging problem has been overlooked, and few studies have emphasized this collateral issue.

The aim of this study was to investigate factors that may be associated with informal caregiver burden for those who provide “elderly-for-elderly care”. We examined the associations among caregiver burden and demographic variables of patients and caregivers, NPS, cognitive impairments, physical dependencies, underlying medical conditions, and contextual factors in a large multicenter cohort of aged caregiver-patient dyads.

## Methods

The National Dementia Registry Study in Taiwan (T-NDRS) is a multicenter research study conducted by the Institute of Population Health Sciences, Taiwan National Health Research Institute since 2017. Eight hospitals (three in northern Taiwan, two in middle Taiwan and three in southern Taiwan) participated in this project. All patients with dementia received clinical examinations, including a thorough survey of medical history, physical and neurological evaluations, laboratory tests (complete blood counts, serum B12 and folic acid, thyroid hormone levels, syphilis serology, routine biochemical tests) and brain image evaluations (computed tomography or magnetic resonance imaging). The T-NDRS aimed to investigate the baseline characteristics (including demographics, cognitive status, and other measures), cognitive and functional changes in patients with dementia and their caregivers’ burden. The T-NDRS study was approved by the ethics committees of the hospital sites.(National Health Research Institutes, Taipei Veterans General Hospital, Kaohsiung Chang Gung Memorial Hospital, Changhua Christian Hospital, Kaohsiung Medical University Hospital, Shuang Ho Hospital, Taichung Veterans General Hospital, National Cheng Kung University Hospital, Cardinal Tien Hospital) Written informed consent and permission for interviews were received from all study participants and their main adult caregivers.

### Study overview and inclusion criteria

**Fig. 1:**
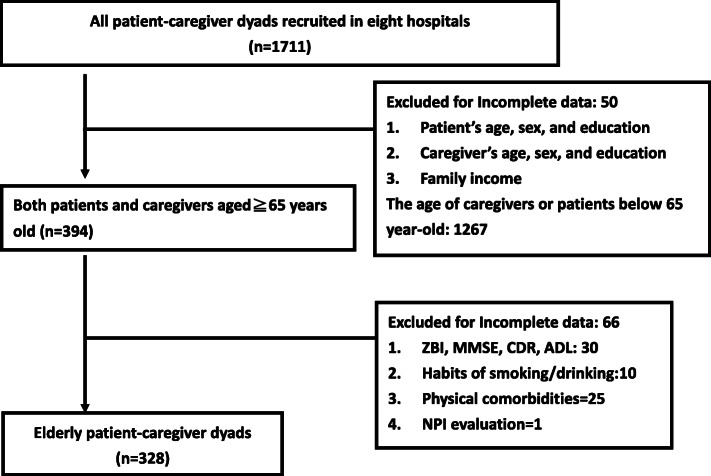
Flow chart of the enrollment of study participants

All participants received assessments, and their family caregivers reported patients’ NPS and their own caregiver burden. General demographics and clinical information, such as history of major psychiatric diseases, neuropsychological and neuropsychiatric disturbances, functional disability, ADLs, and monthly income, physical activity per week were collected [16]. For inclusion, patients in the age range between 65 and 90 years must had a diagnosis of dementia with a clinical dementia rating (CDR) score ≧0.5 (covering from very mild to severe dementia) and had at least one main caregiver defined as the person who frequently took care of/talked to/interacted with the dementia patient for at least 10 h a week. The caregivers should accompany the dementia patients for the interview and annual follow-ups. The exclusion criteria for all participants included having any other central nervous system disease other than dementia, having psychosis not due to dementia, having alcohol use disorder or hepatic encephalopathy, or having expected life expectancy less than 6 months. Only family caregiver-patient dyads aged ≧65 years old were analyzed in this study. From 2017 to 2019, 328 elderly patient-caregiver dyads (19.2%) were retrieved from the T-NDRS database (Fig. 1).

### Diagnostic criteria

The diagnosis of dementia type was made according to any of the following criteria: (1) NIA-AA criteria for Alzheimer’s disease (AD) [17], (2) NINDS-AIREN criteria for vascular dementia [18], (3) Lund-Manchester criteria of frontotemporal dementia [19], (4) 2015 International Dementia with Lewy Bodies (DLB) conference criteria for dementia with Lewy bodies [20], and (5) The Movement Disorders Society (MDS) criteria for dementia from Parkinson’s disease [21].

### Assessment of questionnaire and scores

#### Mini-mental status exam (MMSE)

The MMSE demonstrates moderately levels of reliability and is a most often used assessment instrument for dementia screening. It provides a total score ranging from 0 to 30, with lower scores indicating greater cognitive impairment. It was administered to patients to provide an overall measure of cognitive impairment [22].

#### Clinical dementia rating scale (CDR)

The severity of dementia was defined according to the CDR [23]. The CDR is a widely used clinical staging instrument characterizing the manifestation and severity of dementia, that is generated from a semi-structured interview with the patient and a knowledgeable informant evaluating six areas of cognitive domains (memory, orientation, judgment and problem solving, community affairs, home and hobbies, and personal care). The overall CDR is derived by synthesizing ratings in each of the six domains where score = 0 indicates normal, score = 0.5 signifies uncertain or very mild impairment, and score = 1, 2, or 3 corresponds to mild, moderate, or severe impairment.

#### Neuropsychiatric inventory questionnaire (NPI-Q)

The NPI-Q is a validated caregiver-based questionnaire in which the informants indicated the presence and severity of NPS and caregiver distress in the demented patient during the last few weeks [24]. The informant rates both the severity of the symptoms present on a 3-point scale (1 = mild; 2 = moderate; and 3 = severe) and the associated distress on them using a 5-point scale (0 = no distress; 1 = minimal distress; 2 = mild distress; 3 = moderate distress; 4 = severe distress; and 5 = extreme distress). A total score for the NPI-Q was generated by summing all the individual severity scores.

#### Instrumental activities of daily living scale (IADL)

The Lawton Instrumental Activities of Daily Living Scale was used to evaluate independent living skills such as doing a laundry, telephone using, and dealing with financial matters of the persons with dementia, which consisted of 8 domains of functions [25]. Persons are scored according to their highest level of functioning in that category. A summary score ranges from 0 (low function, dependent) to 8 (high function, independent).

#### Zarit burden interview (ZBI)

The ZBI is a well-known self-report measure of perceived burden among caregivers. The instrument measures the caregiver’s emotion, psychological health, well-being, social and family life, finances, and degree of control over one’s life [26]. This version contains 22 items and each item on the questionnaire question is a statement which the caregiver is asked to endorse on a 5-point Likert scale (0: never; 1: rarely; 2: sometimes, 3: quite frequently; and 4: nearly always).

### Statistical analysis

Categorical variables were presented by numbers with percentages, and continuous variables were presented by means with standard deviations. Chi-square tests and independent t-tests were used to compare categorical and continuous variables, respectively. Multivariable linear regression analysis was conducted to assess the associations between covariates and ZBI scores (caregiver burden). β values and their 95% confidence intervals (95% CIs) were calculated after adjustments in different models. Multivariable linear regression model 1 was adjusted for all variables listed in Table 1 except NPI total scores and NPI severity scores for the 12 items. Model 2 and model 3weremodel 1 plus adjusting for the total summed NPI severity scores or the severity score for each NPI item, respectively. Finally, the approaches of stepwise selection were applied in model 2 and model 3 to identify significant covariables for caregiver burden. All statistical analyses were performed using SPSS version 21.0(IBM, Armonk, NY, USA) with 2-tailed statistical tests. *P* values less than 0.05 were considered statistically significant. We did the power analysis for multiple regression and the power achieved is 99% with sample size of 328.

**Table 1:** Demographic data of patients with dementia (*n* = 328) and their caregivers

	Mean (SD) / n(%)		Mean (SD) / n(%)
Patient_Age	78.82 (6.92)	**Physical diseases**	
Patient_Sex, Male	200 (61.0%)	Hypertension	205 (62.5%)
Patient_Education, yr	9.45 (5.20)	Diabetes	92 (28.0%)
Caregiver_Age	73.65 (6.99)	Hyperlipidemia	101 (30.8%)
Caregiver_Sex, Male	109 (33.2%)	Ischemic stroke	41 (12.5%)
Caregiver_Education,yr	10.08 (4.74)	Hemorrhagic stroke	12 (3.7%)
Relationship		Transient Ischemic Attack	30 (9.1%)
Spouse	285 (86.9%)	Head trauma	17 (5.2%)
Offspring	29 (8.8%)	CAD	17 (5.2%)
Others	14 (4.3%)	Heart failure	7 (2.1%)
		MDD	16 (4.9%)
Live together, yes	300 (91.5%)	Anxiety	23 (7.0%)
Family income		**NPI_severity_Total**	4.45 (5.16)
< 30,000 NTD	104 (31.7%)	NPI severity_Delusion	0.38 (0.81)
30,000 ~ 100,000 NTD	192 (58.5%)	NPI severity_Hallucination	0.31 (0.75)
> 100,000 NTD	28 (8.5%)	NPI severity _Agitation	0.38 (0.77)
MMSE	17.78 (7.07)	NPI severity_Depression	0.39 (0.77)
CDR	1.16 (0.78)	NPI severity_Anxiety	0.40 (0.78)
IADL	4.33 (5.76)	NPI severity_Euphoria	0.08 (0.34)
Drinking, yes	8(2.4%)	NPI severity_Apathy	0.55 (0.91)
Smoking, yes	26 (7.9%)	NPI severity_Disinhibition	0.29 (0.70)
ZBI score	26.65 (18.12)	NPI severity_Irritability	0.48 (0.84)
**Physical activity in 7 days**		NPI severity_Aberrant	0.25 (0.63)
Vigorous activities,yes	29 (8.8%)	NPI severity_ Sleep/nighttime behavior change	0.61 (1.00)
Moderate activities,yes	67 (20.4%)	NPI severity_Appetite	0.32 (0.72)
Walking,yes	234 (71.3%)		
Sitting,yes	327 (99.7%)		

*Abbreviations*: *SD* standard deviation, *NTD* new Taiwan dollar, *MMSE* mini-mental state examination, *CDR* clinical dementia rating Scale, *IADL* instrumental activities of daily living, *ZBI* Zarit Burden Interview, *CAD* coronary artery disease, *MDD* major depressive disorder, *NPI* Neuropsychiatric Inventory

## Results

The demographics and clinical characteristics of the patients and their caregivers are presented in Table 1. A total of 328 patients (61% male, mean age = 78.8, mean education = 9.5 years) were included. Their caregivers were mostly female (66.8%), with a mean age of 73.7 years and a mean education of 10.1 years. The mean ZBI score was 26.7 ± 18.1. The majority of caregivers were spouses (87.0%) or offspring (8.8%). Most caregivers lived with the patients (91.5%) and had earnings in the middle family income class. The caregivers had a similar age distribution to the patients with dementia, with the exception that the caregivers aged over 95 years mostly took care of patients aged 75–84 years (Table 1). Regarding the relationships between the patients and caregivers, the spouses (*n* = 285) were in dyads with patients aged 78.1 ± 6.7 years, and caregivers aged 74.6 ± 7.0 years; the offspring (*n* = 29) were in dyads with patients aged 88.0 ± 2.1 years, and caregivers aged 67.0 ± 1.6 years. With regard to the dementia severity of patients, the average scores were 17.8 on the MMSE, 1.2 on the CDR, and 4.3 on the IADL. Each NPI item demonstrated similar averaged severity with the exception of relatively lower severity of euphoria and relatively higher severity of sleep/nighttime behavior change.

In multivariable model 1(Table 2), the ZBI scores were negatively associated with the highest income class but positively associated with IADLs. Physical diseases, psychiatric disorders such as major depressive disorder and anxiety, physical activities and life habits such as drinking and smoking did not show an influence on the ZBI scores. After the total NPI score was added to the analysis (model 2, Table 2), the NPI total scores were positively associated with increases in the ZBI scores (β: 1.09, 95% CI: 0.68 to 1.49, *p* < 0.001). We further analyzed each NPI item in model 3 (Table 2). The NPI scores for the apathy, depression and irritability domains were significantly associated with ZBI scores. For every additional unit of severity for the aforementioned NPI domains, there was a 3.7-point increase in the ZBI score for apathy (95% CI: 1.5 to 5.9, *p* = 0.001), a 3.3-point increase for irritability (95% CI: 0.3 to 6.3, *p* = 0.03) and a 3.0-point decrease for depression (95% CI:-6.0 to − 0.0, *p* = 0.047). In addition, the NPI anxiety and euphoria domains delivered trend-like positively related influences (Table 2).

**Table 2:** Multivariable linear regressions for ZBI scores of different models

	Model 1		Model 2 (NPI_severity_total)		Model 3(NPI_severity)	
	β(95% C.I)	*P* value	β(95% C.I)	*P* value	β(95% C.I)	*P* value
Pt_Age	−0.34 (− 0.72 to 0.04)	0.080	−0.34 (− 0.71 to 0.02)	0.066	−0.27 (− 0.63 to 0.10)	0.151
Pt_Sex, Male	2.25 (−5.26 to 9.77)	0.555	0.47 (−6.75 to 7.69)	0.899	0.45 (− 6.76 to 7.67)	0.902
Pt_Education, yr	−0.33 (− 0.86 to 0.21)	0.232	− 0.34 (− 0.86 to 0.17)	0.185	−0.37 (− 0.88 to 0.14)	0.157
Caregiver_Age	−0.23 (− 0.62 to 0.16)	0.253	−0.15 (− 0.53 to 0.22)	0.417	−0.24 (− 0.62 to 0.14)	0.211
Caregiver_Sex, Male	−4.99 (− 13.06 to 3.08)	0.225	− 6.58 (− 14.32 to 1.16)	0.095	− 5.62 (− 13.35 to 2.11)	0.153
Caregiver_Education,yr	0.47 (− 0.07 to 1.02)	0.088	0.39 (− 0.13 to 0.92)	0.141	0.32 (− 0.21 to 0.85)	0.241
Relasionship						
Spouse	0.00 (reference)		0.00 (reference)		0.00 (reference)	
Offspring	7.08 (−4.46 to 18.61)	0.228	6.69 (−4.35 to 17.73)	0.234	4.19 (−6.92 to 15.29)	0.459
Others	−5.80 (−16.91 to 5.30)	0.305	−7.79 (− 18.44 to 2.87)	0.151	−7.44 (− 18.17 to 3.30)	0.174
Live together, yes	7.25 (− 1.30 to 15.80)	0.096	4.81 (−3.42 to 13.05)	0.251	3.80 (−4.47 to 12.08)	0.367
Family income						
< 30,000 NTD	0.00 (reference)		0.00 (reference)		0.00 (reference)	
30,000 ~ 100,000 NTD	2.05 (− 2.35 to 6.46)	0.360	2.38 (−1.84 to 6.60)	0.268	2.34 (−1.84 to 6.53)	0.271
> 100,000 NTD	−9.54 (−17.02 to − 2.06)	0.013*	−9.29 (− 16.45 to − 2.14)	0.011*	−8.99 (− 16.08 to − 1.90)	0.013*
MMSE	−0.22 (− 0.65 to 0.22)	0.329	− 0.22 (− 0.64 to 0.19)	0.294	− 0.28 (− 0.70 to 0.14)	0.185
CDR	− 0.75 (−4.47 to 2.97)	0.692	− 2.81 (− 6.45 to 0.83)	0.130	−3.91 (−7.62 to − 0.20	0.039*
IADL	0.96 (0.50 to 1.41)	< 0.001*	0.94 (0.51 to 1.38)	< 0.001*	1.00 (0.55 to 1.45)	< 0.001*
Drinking, yes	−5.18 (− 17.57 to 7.21)	0.411	−3.36 (− 15.23 to 8.51)	0.578	−4.13 (− 15.87 to 7.61)	0.489
Smoking,yes	− 0.84 (− 8.18 to 6.51)	0.823	−1.26 (− 8.29 to 5.77)	0.725	− 0.30 (−7.43 to 6.83)	0.934
**Physical activity in 7 days**						
Vigorous activities,yes	0.63 (−6.60 to 7.87)	0.863	0.71 (−6.21 to 7.63)	0.840	1.58 (− 5.38 to 8.53)	0.656
Moderate activities,yes	−3.20 (−8.67 to 2.27)	0.251	−1.69 (−6.95 to 3.58)	0.528	−1.25 (−6.49 to 4.00)	0.640
Walking,yes	1.23 (−3.68 to 6.14)	0.622	2.29 (−2.43 to 7.00)	0.341	1.30 (−3.52 to 6.13)	0.595
Sitting,yes	7.94 (−26.57 to 42.44)	0.651	−1.75 (−34.95 to 31.45)	0.917	2.65 (−30.83 to 36.13)	0.876
**Physical diseases**						
Hypertension	−1.75 (−5.90 to 2.40)	0.407	−1.03 (− 5.01 to 2.95)	0.611	−1.79 (− 5.76 to 2.18)	0.375
Diabetes	0.87 (−3.50 to 5.23)	0.696	1.52 (−2.67 to 5.70)	0.476	1.93 (−2.32 to 6.19)	0.371
Hyperlipidemia	−0.06 (−4.77 to 4.64)	0.979	0.82 (−3.69 to 5.34)	0.719	0.37 (−4.13 to 4.86)	0.872
Ischemic stroke	0.22 (−6.71 to 7.15)	0.951	0.01 (− 6.62 to 6.64)	0.998	−0.42 (−7.05 to 6.20)	0.900
Hemorrhagic stroke	0.03 (−10.16 to 10.22)	0.995	0.69 (−9.06 to 10.44)	0.889	−1.05 (− 10.76 to 8.66)	0.831
Transient Ischemic Attack	1.09 (−5.71 to 7.88)	0.753	0.91 (−5.59 to 7.42)	0.782	1.38 (−5.08 to 7.83)	0.675
Head trauma	4.01 (−4.81 to 12.84)	0.371	1.44 (−7.05 to 9.94)	0.738	−0.97 (−9.71 to 7.77)	0.828
CAD	2.58 (−6.35 to 11.52)	0.570	1.94 (−6.62 to 10.49)	0.656	2.89 (−5.84 to 11.61)	0.516
Heart failure	−3.76 (−17.79 to 10.27)	0.599	−2.59 (−16.02 to 10.84)	0.704	−5.97 (− 19.40 to 7.46)	0.382
MDD	−1.34 (− 13.32 to 10.65)	0.826	−5.62 (− 17.20 to 5.95)	0.340	−6.08 (− 17.90 to 5.73)	0.312
Anxiety	6.54 (−3.58 to 16.66)	0.205	3.37 (−6.38 to 13.13)	0.496	5.32 (−5.12 to 15.75)	0.317
**NPI_Total**			1.09 (0.69 to 1.49)	< 0.001*	N/A	
NPI_Delusion					1.26 (−1.57 to 4.10)	0.380
NPI_Hallucination					−2.03 (−5.21 to 1.15)	0.211
NPI_Agitation					0.99 (−2.09 to 4.08)	0.526
NPI_Depression					−3.03 (−6.00 to −0.06)	0.046*
NPI_Anxiety					2.84 (−0.16 to 5.84)	0.063
NPI_Euphoria					5.82 (−0.28 to 11.91)	0.061
NPI_Apathy					3.70 (1.47 to 5.93)	0.001*
NPI_Disinhibition					0.63 (−2.83 to 4.09)	0.721
NPI_Irritability					3.33 (0.30 to 6.36)	0.031*
NPI_Aberrant motor acitivity					−0.83 (−4.35 to 2.70)	0.644
NPI_Night					1.25 (−0.72 to 3.22)	0.214
NPI_Appetite					1.97 (−0.76 to 4.69)	0.157

*Abbreviations*: *NTD* new Taiwan dollar, *MMSE* mini-mental state examination, *CDR* clinical dementia rating Scale, *IADL* instrumental activities of daily living, *CAD* coronary artery disease, *MDD* major depressive disorder

*p < 0.05

In the stepwise multivariable regression (Table 3), the model that included total NPI severity scores showed that IADLs, the highest class of family income, caregiver age and total NPI severity scores were significantly correlated with the ZBI scores. In detail and listed by the order of importance, another model including the 12 NPI items demonstrated that IADLs, NPI_irritability/lability, NPI_apathy/indifference, the highest family income, and caregiver age had significant influences on the ZBI scores. Other NPI domains did not have a significant impact on caregiver burden.

**Table 3:** Multivariable linear regression in a stepwise manner

NPI_Severity_total model	β (95% C.I)	*P* value
NPI_Severity_Total	1.03 (0.68 to 1.37)	< 0.001
IADL	0.79 (0.48 to 1.10)	< 0.001
Family income: > 100,000 NTD vs < 30,000 NTD	−10.50 (−16.74 to − 4.26)	0.001
Caregiver_Age	−0.40 (− 0.65 to − 0.15)	0.002
**NPI_severity_12 items model**		
IADL	0.83 (0.52 to 1.13)	< 0.001
NPI_irritability_severity	4.25 (2.11 to 6.39)	< 0.001
NPI_Apathy_severity	3.83 (1.85 to 5.81)	< 0.001
Family income: > 100,000 NTD vs < 30,000 NTD	−10.92 (−17.06 to −4.77)	0.001
Caregiver_Age	−0.41 (−0.65 to − 0.16)	0.001
NPI_Euphoria_severity	5.76 (0.62 to 10.90)	0.028

*Abbreviations*: *IADL* instrumental activities of daily living, *NPI* Neuropsychiatric Inventory

## Discussion

This nationwide multicenter study included informal caregivers of patients who, for the most part, suffered from mild or moderate probable or possible AD and exhibited, on average, mild NPS (Table 1). The study was performed in 8 memory clinics of medical centers and local hospitals in both urban and rural areas. Among our aged patient-caregiver dyads, most patients were male and cared for by female family caregivers. This corresponds to international findings that have shown that caregiving for dementia patients is usually informal and a female dominant [9, 27–31]. Spouses played a major role (86.9%) in caregiving in these old-old dyads, rather than offspring, such as middle-aged daughters or daughters-in-law [32, 33].

We found that family monthly income, IADL functional impairment, NPS, and caregiver age emerged as independent predictors of elderly caregiver burden in the present cohort regarding after adjusting for severity of dementia, medical comorbidities, education, relationship between patients and caregivers, and physical activities per week. Many studies have consistently found that both NPS and functional impairment caused more distress to family caregivers than other care demands, such as cognitive deficits [8].

Our findings corroborate previous research that IADL deficits in patients with dementia were associated with more caregiver burden and depression [34, 35].In addition to the physical burden of the caregivers, the ADL dependency of the patient is also correlated with the number of care hours, and it has been shown to be the only factor independently associated with missing more hours at work for those who were employed [29].

By conducting multiple regression analysis using different NPS as individual predictors of caregiver burden, we found that the NPI apathy and NPI irritability subscores were independent factors for caregiver burden. In a prior study of 548 French caregivers without mention of the mean value of caregivers’ age, it found that apathy, agitation/aggression, aberrant were related to caregivers’ burden significantly [34]. Other studies recruited older adult caregivers mainly showed that delusions, agitation/aggression and irritability of demented patients caused the most distress to their caregivers [10, 36]. Behavioral and psychological symptoms of dementia may worsen the caregiver’s burden of providing care for ADLs [37]. Resistiveness to care is common in demented patients especially those residing at home, Fauth and colleagues found that NPS that observed in the context of ADL assistance (i.e., care refusal) mainly accounted for the relation between ADL dysfunction and level of caregiver burden [38].

Dementia creates a substantial burden on human and financial resources, and the costliest part of the total cost of home care is unpaid assistance [39]. It is common for family caregivers to experience financial strain as a result of providing care during the long disease course of dementia, both when the relative is cared for at home or in nursing homes [40]. Adult caregivers with low incomes perceive more distress than caregivers with higher incomes [11, 41], and low levels of financial resources have been shown to predict depressed mood in caregivers of persons with dementia [42]. Caregivers with higher financial resources probably have more access to supportive services (home health aides, adult day care) that may reduce their burden [42]. Compared with adult caregivers, most elderly caregivers are retired or unemployed. The government launched he Long-Term Care 2.0 (LTC2.0) Plan in 2016 and the payment system was categorized into four parts as personal care, professional care, transportation assistive devices and barrier free environment modification, and respite care for family caregivers. Each recipient has an upper limit and care delivery is paid by fee-for-service. Although co-payments are exempted for extremely-low-income users, others are responsible for fee for service usage exceeding the upper limit with the co-payment rate of 16% under current LTC2.0 system [43]. Developing policies such as programs that pay family caregivers, providing financial compensation, and special tax deductions for caregivers may be helpful. Further larger-scale follow-up cohort studies are required to verify this opinion.

Similar to several publications [27, 30, 44, 45] but in contrast to other studies [46–48], our study revealed an inverse relationship between caregiver burden and caregiver age among those aged 65 and older. Young-old caregivers may still be at work, and caregiving-related contradiction with their job and leisure activities might explain the more severe burden in young-old than old-old caregivers.

Our findings may help to identify elderly caregivers who are at risk of caregiver burden and serve to take targeted steps to alleviate their suffering, such as effective management of IADL functional decline and NPS of the client, personal and psychological support for the caregiver, and presumably, better financial funding of informal care.

This study had several limitations. First, information about caregiver-related comorbidities and general health conditions, social support, and cumulative duration of care was not available in the T-NDRS. Without these, we cannot evaluate their impacts. Family caregivers of person with dementia reported a greater number of physical health problems and worse overall health compared with non-caregiver controls [49]. Since we didn’t evaluate the underlying diseases of family caregivers (such as diabetes, arthritis, cardiovascular disease, poor immune functioning) which may contribute to caregiver burden, the present study could not go further to explore the influence between caregiver’s general health and burden. Second, this study only recruited family caregiver-patient dyads who had sought medical services. Although different types of caregivers may have different level of perceived burden, previous studies found that no matter whether the background of the caregiver is family, professional, or paid caregiver, the most relevant burden is psychological burden and stress caused by behavioral disturbances [50, 51]. In addition, most of the eight participating hospitals were medical centers, and our findings may not be generalizable to those visiting community clinics alone or those not seeking medical help.

## Conclusion

Older caregivers of aged demented patients experience higher caregiver burden, which may be positively associated with impairments in functional autonomy and more severe NPS of apathy and irritability and negatively correlated with higher family income.

## Data Availability

The data that support the findings of this study are available from National Dementia Registry Study group (T-NDRS) but restrictions apply to the availability of these data, which were used under license for the current study, and so are not publicly available. Data are however available from the authors upon reasonable request and with permission of T-NDRS study group (National Health Research Institutes, Taiwan) via corresponding author.

## Abbreviations

AD: Alzheimer’s disease
ADL: Activities of daily living
CDR: Clinical dementia rating
DLB: Dementia with Lewy Bodies
IADL: Instrumental Activities of Daily Living Scale
MDS: Movement Disorders Society
MMSE: Mini-mental Status Exam
NPI-Q: Neuropsychiatric Inventory Questionnaire
NPS: Neuropsychiatric symptoms
T-NDRS: National Dementia Registry Study in Taiwan
ZBI: Zarit Burden Interview
